# 

**Published:** 1858-06

**Authors:** 


### CONTENTS.

ORIGINAL COMMUNICATIONS— PAGE.

Surgical Cases. By C. Brackett, M. D. .......... 245

Observations on the Febrile Movement in Disease. By J. R. Hayes, M. D. .......... 252

Cases in Practice. By L. H. Angell, M. D. .......... 258

BOOK AND PAMPHLET NOTICES—

Materia Medica and Therapeutics. By Thos. D. Mitchell, A.M., M.D., 261

Contributions to Operative Surgery and Surgical Pathology. By J. M. Carnochan .......... 263

Preserving Bodies for Dissection. By Bennet Dowler, M. D .......... 264

EXTRACTS—

American Medical Association—Eleventh Annual Meeting .......... 266

PROCEEDINGS OF MEDICAL SOCIETIES—

Illinois State Medical Society .......... 281

Indiana State Medical Society .......... 285

